# Correction: Kang et al. Preparation and In Vivo Evaluation of a Lidocaine Self-Nanoemulsifying Ointment with Glycerol Monostearate for Local Delivery. *Pharmaceutics* 2021, *13*, 1468

**DOI:** 10.3390/pharmaceutics14010155

**Published:** 2022-01-10

**Authors:** Ji-Hyun Kang, Kwang-Hwi Yoo, Hyo-Young Park, Seung-Min Hyun, Sang-Duk Han, Dong-Wook Kim, Chun-Woong Park

**Affiliations:** 1 College of Pharmacy, Chungbuk National University, Cheongju 28160, Korea; jhkanga@naver.com (J.-H.K.); dbrhkdgnl1@naver.com (K.-H.Y.); 2 DONG-A Pharm. Co., Ltd., Yongin 17073, Korea; Hyogoo312@donga.co.kr (H.-Y.P.); hsm@donga.co.kr (S.-M.H.); tilldie@donga.co.kr (S.-D.H.); 3 Department of Pharmaceutical Engineering, Cheongju University, Cheongju 28503, Korea

The authors wish to make the following corrections to this paper [1].

In the original publication, there was a mistake in the legend for Table 1 as published, dl-Methylephedrine HCl of L4 is “0.5” and Petrolatum of L4 is “91.45”. The corrected Table 1 appears below

There was an error in the original article in Figure 1, “Lidcocaine” is misspelled as a legend. The correct Figure 1 appears below.

There was an error in the original article in Figure 2, “Lidcocaine” is misspelled as a legend. The correct Figure 2 appears below.

The authors apologize for any inconvenience caused and state that the scientific conclusions are unaffected. The original publication has also been updated.

## Figures and Tables

**Figure 1:**
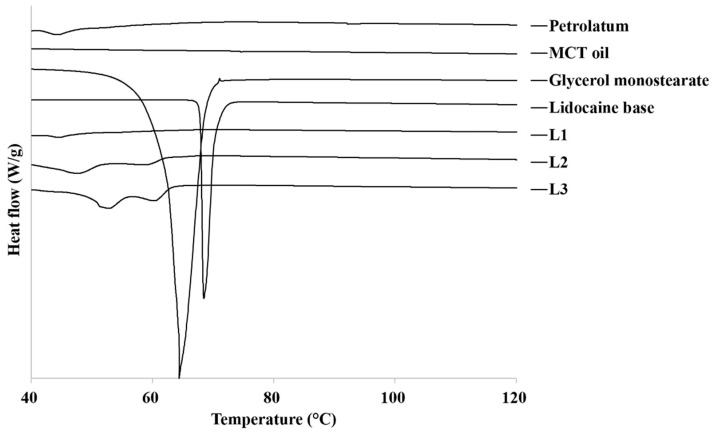
DSC thermograms of raw materials and prepared lidocaine ointments. MCT, medium-chain triglyceride.

**Figure 2:**
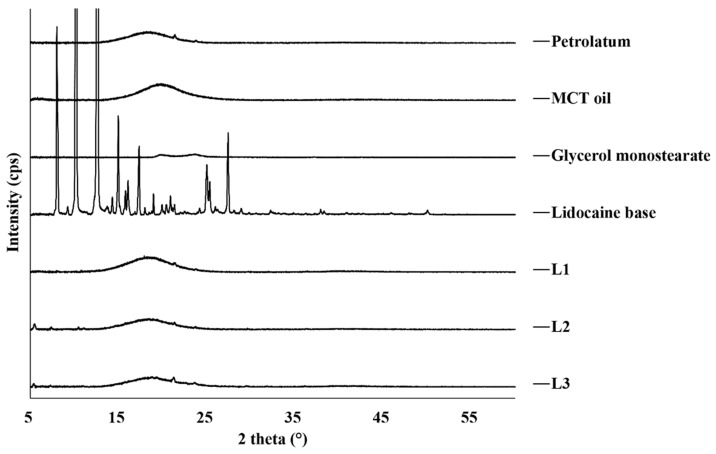
X-ray diffraction patterns of raw materials and prepared lidocaine ointments. MCT, medium-chain triglyceride.

**Table 1:** Formulation and rheological characterization of lidocaine ointments.

Excipient.	L1	L2	L3	L4	L5	L6
Petrolatum	95.00	91.00	86.00	91.95	86.45	81.45
MCT oil	1.00	1.00	1.00	1.00	1.00	1.00
Glycerol monostearate	-	5.00	10.00	-	5.00	10.00
Vitamin E-acetate	-	-	-	3.00	3.00	3.00
Lidocaine base	3.00	3.00	3.00	3.00	3.00	3.00
Allantoin	-	-	-	1.00	1.00	1.00
Prednisolone acetate	-	-	-	0.05	0.05	0.05
dl-Methylephedrine HCl	-	-	-	-	0.5	0.5
pH	8.4	8.4	8.5	8.5	8.5	8.5
Viscosity (×10^3^ cP)	-	-	-	23.5 ± 0.5	41.9 ± 0.8	95.2 ± 0.9
Minimum extrusion force (N)	-	-	-	19.5 ± 0.2	25.2 ± 0.8	54.9 ± 0.9

Abbreviations: MCT, medium-chain triglyceride, HCl, hydrochloride.
